# The New Marriage Law

**Published:** 1913-09

**Authors:** 


### Department of Eugenics.

Conducted by Dr. Malone Duggan and Dr. Theo. Y. Hull.

### THE TEXAS STATE SOCIETY OF SOCIAL HYGIENE.

Headquarters: San Antonio, Texas.

DR. MALONE DUGGAN, President, San Antonio.
DR. F. E. DANIEL, 1st Vice President, Austin.
PROF. A. CASWELL ELLIS, 2d Vice President, Austin.
MRS. ELI HERTZBERG, 3d Vice President, San Antonio.
DR. THEO. Y. HULL, Secretary, San Antonio.
MR. W. L. EVANS, Treasurer, San Antonio.

EXECUTIVE COMMITTEE.

Dr. Malone Duggan, Chm.
Dr. Theo. Y. Hull, Sec'y.
Dr. W. F. McCaleb.
Hon. C. H. Jenkins.
Rev. A. W. S. Garden.
Hon. J. C. Willacy.
Mr. W. L. Evans.
Dr. Frank D. Boyd.
Dr. J. R. Nichols.
Dr. J. M. McCutchan.
Dr. W. A. King.
Dr. J. W. Torbett.
Dr. F. C. Walsh.
Dr. Joe Reuss.
Dr. Frank Paschal.

### The New Marriage Law.

Reports from Pittsburgh, a city which has furnished some spicy chapters to the record of America's divorce records, indicate that much resentment has been aroused against the new State law safeguarding the marriage contract.

Thirty couples in that city recently declined to answer the questions required by the new law. They left the license bureau promising to go into other States where no such inquisitive laws were enforced.

The incidents were distressing to the parties involved. To be denied entrance to the gate of matrimony because of some mental or physical taint was something to fume over and denounce as an unwarranted interference with personal liberty.

Instead of proving the law unnecessary and without reason these thirty cases are the strongest arguments that could be advanced of the need for such restrictions. A clean marriage statute in all the States would lessen the records of crimes and morbidity, which prove so costly.

While physical soundness is not the sole requisite for happy marriage, the records of eugenics prove what results from the union of persons afflicted with mental and moral and physical defects may accrue. No one questions the immediate effects of the union of unsound persons, and the criminal records show how the poison spreads out in future generations.

The "conspiracy of silence" which permits the contamination of the race to continue is largely responsible for this unhappy situation. There should be more plain talk in legislative bodies.—*Atlanta Constitution.*

